# Inhibition of myostatin prevents microgravity-induced loss of skeletal muscle mass and strength

**DOI:** 10.1371/journal.pone.0230818

**Published:** 2020-04-21

**Authors:** Rosamund C. Smith, Martin S. Cramer, Pamela J. Mitchell, Jonathan Lucchesi, Alicia M. Ortega, Eric W. Livingston, Darryl Ballard, Ling Zhang, Jeff Hanson, Kenneth Barton, Shawn Berens, Kelly M. Credille, Ted A. Bateman, Virginia L. Ferguson, Yanfei L. Ma, Louis S. Stodieck

**Affiliations:** 1 Lilly Research Laboratories, Indianapolis, Indiana, United States of America; 2 Dept. of Aerospace Engineering Sciences, BioServe Space Technologies, University of Colorado, Boulder, Colorado, United States of America; 3 Dept. of Biomedical Engineering, University of North Carolina, Chapel Hill, North Carolina, United States of America; 4 TechShot, Inc., Greenville, Indiana, United States of America; 5 Dept. of Mechanical Engineering, University of Colorado, Boulder, Colorado, United States of America; Tohoku University, JAPAN

## Abstract

The microgravity conditions of prolonged spaceflight are known to result in skeletal muscle atrophy that leads to diminished functional performance. To assess if inhibition of the growth factor myostatin has potential to reverse these effects, mice were treated with a myostatin antibody while housed on the International Space Station. Grip strength of ground control mice increased 3.1% compared to baseline values over the 6 weeks of the study, whereas grip strength measured for the first time in space showed flight animals to be -7.8% decreased in strength compared to baseline values. Control mice in space exhibited, compared to ground-based controls, a smaller increase in DEXA-measured muscle mass (+3.9% vs +5.6% respectively) although the difference was not significant. All individual flight limb muscles analyzed (except for the EDL) weighed significantly less than their ground counterparts at the study end (range -4.4% to -28.4%). Treatment with myostatin antibody YN41 was able to prevent many of these space-induced muscle changes. YN41 was able to block the reduction in muscle grip strength caused by spaceflight and was able to significantly increase the weight of all muscles of flight mice (apart from the EDL). Muscles of YN41-treated flight mice weighed as much as muscles from Ground IgG mice, with the exception of the soleus, demonstrating the ability to prevent spaceflight-induced atrophy. Muscle gene expression analysis demonstrated significant effects of microgravity and myostatin inhibition on many genes. Gamt and Actc1 gene expression was modulated by microgravity and YN41 in opposing directions. Myostatin inhibition did not overcome the significant reduction of microgravity on femoral BMD nor did it increase femoral or vertebral BMD in ground control mice. In summary, myostatin inhibition may be an effective countermeasure to detrimental consequences of skeletal muscle under microgravity conditions.

## Introduction

The microgravity conditions of spaceflight are known to result in a rapid atrophy of skeletal muscle and also a loss of underlying bone [1,2]. The decrease of muscle mass leads to weakness and diminished functional capacity, particularly noticeable in astronauts upon return to gravity condition [3–6]. Rodents have proven to be a good animal model for muscle loss in space. Lalani *et al*. [7] showed that rats flown on the Space Shuttle (STS-90) for 17 days lost 5% of body weight and muscle weights (quadriceps, gastrocnemius, biceps, tibialis) were 19–24% lower in weight than their ground controls. In 9-week-old (i.e., skeletally immature) female C57Bl/6 mice flown on the Space Shuttle (STS-108, -135) for ~13 days, both body weight and muscle mass (soleus, gastrocnemius, plantaris) were diminished where atrophy was indicated by decreased myofiber cross-sectional area as compared to ground controls [8–10]. Decreased myofiber CSA of soleus and EDL muscles was observed in skeletally mature 19–20 week-old male C57Bl/6 mice flown on the BION-M1 biosatellite for 30 days [11]. The femoral quadriceps muscle group from these same mice also showed significant atrophy and myofibril degeneration [12]. Long-term exposure to microgravity conditions of 91 days aboard the International Space Station (ISS) interestingly showed no effect on myofiber CSA of the EDL but a pronounced 35% decrease in soleus CSA [13], confirming that the type I soleus appears particularly prone to wasting. In mice flown on the 30 day Bion-M1 mission, both fore- and hindlimb grip strength was significantly reduced [10]. While direct biomechanical force measurements in murine muscle following microgravity exposure is limited, the tibialis anterior and masseter muscle fibers showed a correlation of decreased function with increased atrophy [14]. With regards to effects of space on murine bones, a number of reports have described diminished strength, microarchitecture, and mechanical properties following exposure of mice and rats to microgravity [15–19].

Skeletal muscle atrophy not only occurs during the unloading conditions of spaceflight, but is also associated with many disease conditions on Earth such as cancer cachexia, muscular dystrophy, sporadic inclusion body myositis, as well as with advanced aging (i.e., sarcopenia) [20]. Loss of muscle mass results in reduced muscle function and this can have a direct negative impact on functional performance such as the ability to carry out activities of daily living [21]. Muscle wasting has also been associated with poor prognosis and mortality [21]. While a treatment for spinal muscle atrophy was approved in 2016 for clinical use in the United States [22], medications that treat or prevent muscle atrophy remain largely unavailable. Exercise and/or physical therapy remain the only options to counteract this condition. Astronauts in space perform resistance exercises several hours a day to combat muscle wasting and bone loss, which is both inefficient and insufficient [4,23]. With future goals of manned spaceflight to the moon and Mars, especially if exercise equipment is limited, the ability to carry out missions may be compromised. On Earth compliance is also an issue for the ill and elderly. Fortunately, a number of experimental medicines to treat muscle atrophy are making their way through clinical trials. Many of these therapies have targeted the growth factor myostatin (also known as GDF8) or its signaling pathway (reviewed in Smith and Lin, 2013) [24]. Myostatin is a highly conserved secreted protein belonging to the TGFβ superfamily of growth factors. Myostatin is a negative regulator of vertebrate skeletal muscle mass as demonstrated by the increased muscle mass and function seen in animals lacking functional copies of this growth factor [24]. Genetic myostatin deficiency is also associated with increased bone cross-sectional area, bone density and strength [25]. LY2495655 is an anti-myostatin antibody that has been shown to cause an increase in muscle mass and function in mice [26]. A Phase 2a clinical trial in the weak elderly demonstrated that inhibiting myostatin with LY2495655 was able to not only increase appendicular lean body mass by 0.43 kg over the course of the 24-week trial, but also to significantly improve stair climbing time, chair rise with arms, and fast gait speed [27].

The goal of the current study was to determine if myostatin inhibition could prevent the long-term microgravity-induced muscle atrophy expected in mice kept on board the International Space Station. For the first time in spaceflight, and onboard the ISS, measures of skeletal muscle function as well as lean mass were collected *in vivo* on orbit at both interim as well as terminal timepoints. In addition, further characterization of the effect of muscle and bone unloading that occured in space was conducted. The results confirm and extend the characterization of the loss of muscle and bone induced in mice by spaceflight. In addition, a myostatin antibody was able to prevent the loss of both muscle mass and function observed in microgravity. Not only do these results provide foundational preclinical data to support potential therapeutic intervention during long-duration space missions, but they also provide positive results of myostatin inhibition in a unique and useful global animal model of muscle wasting not possible on Earth.

## Materials and methods

### Antibodies

YN41 (also called LSN2478185) is an anti-myostatin (GDF8) mouse IgG1 antibody that was derived from injecting mice with full length mature myostatin with properties as described in Smith et al. (2015) [26]. Control antibodies were IgG1 antibodies with known antigen binding generated within Eli Lilly and Company. Antibodies were kept in long-term storage at -80°C, thawed prior to use and diluted with 1× PBS pH 7.4 (Invitrogen, Gibco) and stored at 4°C for the duration of the study. Pharmacokinetic and dynamic analysis of YN41 in mice supports weekly dosing at 10 mg/kg [26] However, in order to reduce astronaut time, dosing was performed every two weeks at 20 mg/kg with similar coverage and response.

### Care and use of laboratory animals

All animal studies were conducted in strict accordance with the American Association for Laboratory Animal Care institutional guidelines. All *in vivo* experimental protocols were approved by both the NASA flight Institutional Animal Care and Use Committee (IACUC) based at NASA Ames Research Center (Moffett Field, CA) and at the NASA Kennedy Space Center (Protocol CAS-15-01-Y1).

### Mice and live phase operations

Female BALB/cAnNTac mice were acquired from Taconic Farms (Germantown, NY) and shipped to Kennedy Space Center (KSC) Animal Care Facilities at 8 weeks of age. Mice were assessed for specific pathogen free (SPF) compliance prior to shipment from Taconic, within a week of arrival at NASA KSC and at 10 days before launch (L-10 days). After arrival at KSC, mice were adapted to spaceflight cage conditions including wire-mesh floors, spring operated Lixit drinking mechanism and NASA-provided nutrient food bar diet [28]. At 1 week prior to launch mice were caged at double density of n = 10 mice/cage to adjust to launch housing conditions. Fifty mice were selected for inclusion in the study and assigned to equal-sized groups: Baseline, Ground IgG, Ground YN41, Flight IgG, Flight YN41. Body weights across these groups, which were 12 weeks of age at launch, averaged 19.3–19.8 g with a standard deviation of 0.7 g.

At L-4 to L-2 days, baseline measures were collected on all animals including forelimb grip strength and body composition by DEXA. Baseline controls (n = 10) were sacrificed on L+1 and tissue collection carried out as described. One day prior to launch, flight and ground control animals were injected subcutaneously (SQ) with control IgG (Flight IgG) or YN41 myostatin antibodies (Flight YN41) at 20 mg/kg in a total dosing volume of 200 μl prior to loading into the transportation habitat. YN41 treated mice were loaded into one side of the Transporter housing system [29] and IgG mice into the other side (n = 10 mice/side); ground controls were similarly loaded into a NASA mouse Transporter. The Transporter habitat was loaded into the Space-X Dragon capsule and launched into low Earth orbit on the CRS-8 mission on April 8^th^, 2016. The capsule berthed with the ISS at 41 hours post-launch, and approximately 90 hours post launch mice were transferred to 2 ISS Habitats with each having 2 compartments (i.e., sides) that housed 2 controls and 3 treated animals and vice-a-versa. Mice groups were further dosed with YN41 myostatin antibodies or control IgG antibodies at 20 mg/kg in 200 μl at 2 and 4 weeks post-launch.

The ground control groups (Ground IgG, Ground YN41) were treated as identically as possible to flight animals, including housing in the same Transporter and Habitat housing systems. Ground controls were offset from flight animals by 3 days (L+3) to enable exposure to matched environmental conditions (i.e., temperature, humidity and carbon dioxide levels).

At 4 weeks post-launch, forelimb grip strength and body composition via Dual Energy X-ray Absorptiometry (DEXA) were collected over a period of 3 days (note: this extended duration is due to limited astronaut crew time on the ISS). At 6 weeks post-launch and 2 days prior to termination, grip strength was again assessed. Body composition measures via DEXA were collected just prior to termination. For termination, both ground control and flight mice were anesthetized and subjected to cardiac puncture followed by cervical dislocation.

Following termination, the skin was removed from the right hindlimb and the entire limb detached at the pelvis and immersion fixed in 10% neutral buffered formalin and stored at room temperature. The remaining whole carcass was stored at -80°C (ground) or -95°C (flight) until time of dissection. Flight samples were returned to Earth on SpX-9 on August 26, 2016. All additional tissues and organs were collected and processed from both control and flight frozen carcasses as outlined below.

### Grip strength measures

Forelimb grip strength measures [30] were acquired with a digital Grip Strength Meter (Columbus Instruments Model 0167-004L). Identical instruments were used to assess flight mice on the ISS and ground controls. At each time point (0, 2, 4, and 6 weeks post launch), four-limb measurements were acquired in the compression peak (C PEAK) mode. A total of 4 readings were recorded from each mouse with the lowest and highest readings excluded and the remaining values averaged.

### *In vivo* body composition measures

Body composition was measured by DEXA using a Lunar PIXImus (GE Lunar Corp, WI) or a derivative thereof modified for spaceflight and known as the ISS Bone Densitometer (Techshot, Greenville, IN, USA). Mice were anesthetized for pre-flight data collection through isoflurane inhalation (baseline and pre-flight animals). During the mission, two body composition measurements were obtained from flight and ground control animals. For the first intermediate time point (4 weeks after launch) and to accommodate the need to anesthetize mice on the ISS, mice were injected IP with ketamine/xylazine (80/15 mg/kg, flight and ground controls). Upon completion of DEXA measurements, mice were administered atipamezole IP (1 mg/kg), placed individually into a warming device (36°C) and allowed to fully recover before being returned to their Habitats. At termination, mice were anesthetized with ketamine/xylazine/acepromazine (120/15/3 mg/kg) and DEXA measures collected prior to cardiac puncture.

**Fig 1:**
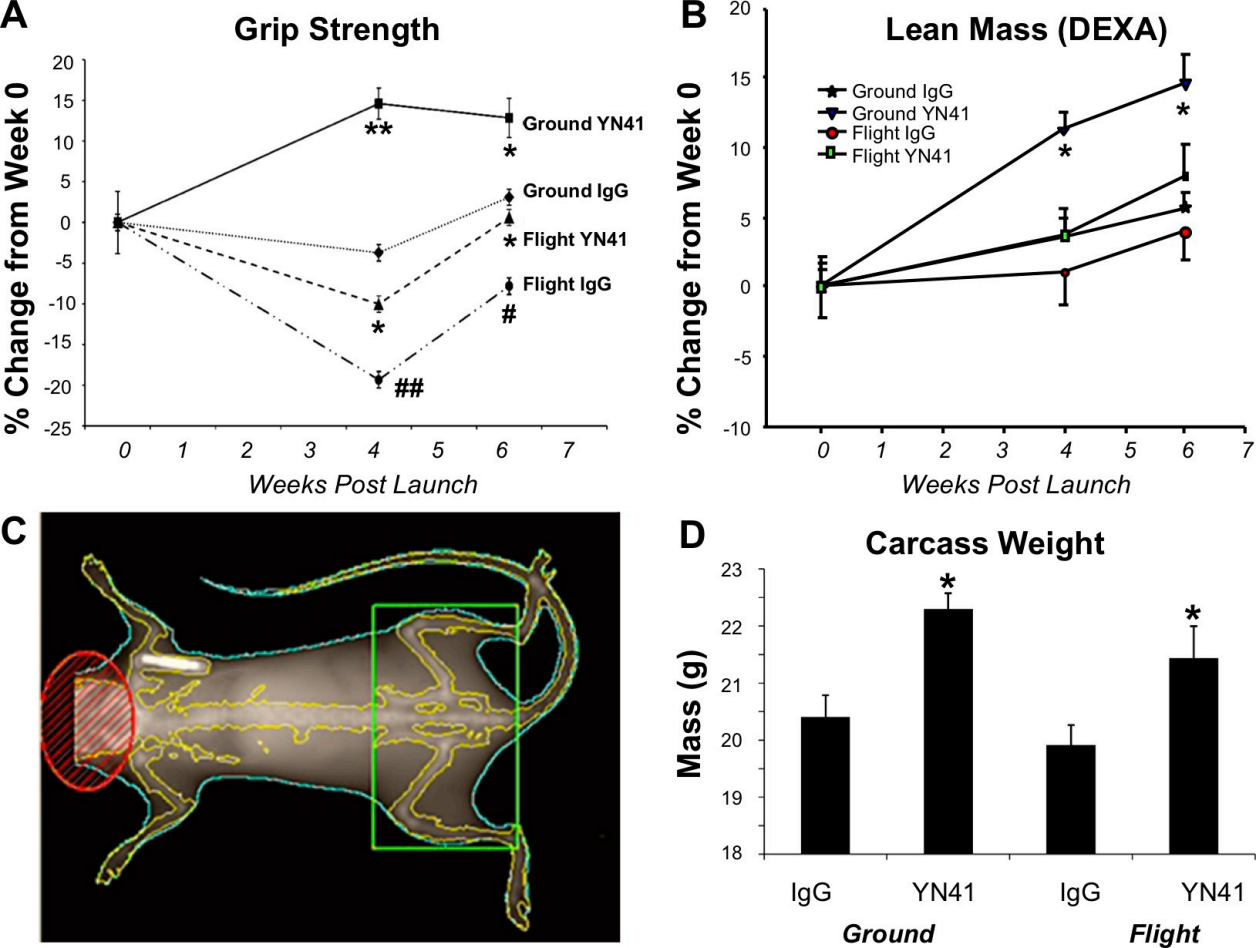
Effect of myostatin inhibition on muscle function and lean muscle mass under conditions of microgravity. Grip strength (A) and lean muscle mass (B) were measured in Ground IgG, Ground YN41, Flight IgG and Flight YN41 groups at launch and weeks 4 and 6 post-launch, expressed as percent change from the same groups measured at baseline, with all baseline measurements having been made on the ground using Lunar PIXImus. (C) Representative mouse image from the DEXA densitometer showing the region of interest (ROI) of the hindlimb outlined in black used in panel B. (D) Carcass weights of all groups. Significance (p < 0.05 *, 0.001 **) for comparison to respective control IgG group or to Ground IgG group (p < 0.05 #, p < 0.001 ##) is noted.

DEXA-based bone mineral density (BMD) and lean mass values were based on a region of interest (ROI) centered on the hindlimb and hip regions. The ROI was established between the ankle joint and the iliac crest (6^th^ lumbar vertebra) and between the outermost regions of the left and right hindlimbs (Fig 1C). For BMD measurements the correction factor between the ISS Bone Densitometer and the ground-based Lunar PIXImus was determined to be 1.000 ± 0.009 so the data was uncorrected. Lean body mass was also derived from DEXA data, in which case the correction factor from the ISS Bone Densitometer to the Lunar PIXImus was determined to be 0.928 ± 0.010, which was applied to the lean body mass determined during space flight.

### Body weight and tissue collection

Carcasses were carefully thawed, the left hindlimb was immediately removed, and the gastrocnemius, quadriceps, tibialis anterior, soleus, plantaris and extensor digitorum longus (EDL) muscles were collected, weighed and placed in RNALater. Bones were collected and cleaned of non-bony tissue, and stored in 70% ethanol at room temperature for subsequent analyses. Heart and brain weights were also collected. Body weights of all mice did not include the skin of the right hindlimb.

### Muscle and bone histology

After fixation and return to Earth, the lower hindlimb muscle bundle (i.e., containing gastrocnemius, soleus and plantaris) was dissected from the right hindlimb and bisected to obtain cross sections of the muscle fibers. The bundle was trimmed at three levels: proximal, mid-muscle bundle and distal. The trimmed muscle samples were routinely processed and embedded in paraffin, sectioned at 4 μm, and stained with hematoxylin and eosin and a histochemical stain, Chandler’s reticulum, to highlight the muscle fiber basement membrane for image analysis. The intact pelvic limbs were routinely decalcified. After decalcification the femur was carefully disarticulated from the coxofemoral joint and was transected proximal to the femoral-tibial joint, routinely processed and paraffin embedded with a longitudinal orientation to allow evaluation of the femoral head and articular cartilage. The tibia was transected below the femoral-tibial joint in order to isolate the joint. The joint was bisected along the frontal plane, routinely processed and the cut surface embedded in paraffin so that the exposed center of the joint was sectioned to allow evaluation of articular cartilage and other joint tissues.

The bone sections were stained with toluidine blue. The stained slides for all muscle and bone samples were examined by a pathologist. The reticulum-stained sections of the gastrocnemius mid-bundle were digitized for image analysis using an Aperio Scan Scope AT2 (Leica Biosystems).

### Muscle fiber quantitation

Muscle fiber cross-sectional area was measured in the scanned images using the Halo software package MuscleFiber module (MuscleFiber v2.2.1, HALO v2.0 1145.31) (Indica Labs). Briefly, each entire reticulum-stained gastrocnemius bundle section was outlined in the Halo software. Histology artifacts (section tears, wrinkles), anatomic structures that interfered with muscle fiber recognition (tendons, oblique fibers) and poorly detected muscle fibers were removed manually from analysis using the software exclusion tool. The MuscleFiber module identified and measured all of the remaining fibers in the bundle, reporting the total fiber count and average fiber area. The average fiber area was used for comparison across groups. One entire mid-bundle section was quantified per mouse averaging 8186 fibers per section (ranging from 3828–12140 fibers per section).

### *Ex vivo* bone measures and biomechanics

Femora and L5 vertebrae were analyzed by quantitative microcomputed tomography (microCT) at two length scales using, first, a LaTheta LTC-100 CT scanner (Aloka; 100 μm voxels) [31]. Briefly, a scan of each femur was performed at both 0.4 and 4.4 mm from the end of the growth plate for analysis of the distal metaphysis and midshaft, respectively. The femur metaphysis contains both trabecular and cortical bone whereas the midshaft femur contains only cortical bone. Aloka software (SYS-C320 version 1.5) was used to assess BMC and bone area for the entire cross-section of both trabecular and cortical bone as in Smith *et al*. (2012) [31]; area BMD was calculated as BMC normalized to bone volumetric area. The left, distal femur was next evaluated for cortical and trabecular bone microarchitecture [32] (μCT80; Scanco Medical AG, Bruttisellen, Switzerland; 70 kVp, isotropic voxel size of 10 μm). Volumes analyzed were located at the distal femur metaphysis, starting 0.5 mm proximal to the epiphyseal line and extending 1 mm proximally. Trabecular and cortical bone were each independently segmented and analyzed using SCANCO evaluation software according to established guidelines [33]. Measured trabecular bone parameters included bone volume fraction (BV/TV), trabecular bone number (Tb.N), trabecular thickness (Tb.Th), trabecular separation (Tb.Sp), and trabecular volumetric bone mineral density (vBMD). Cortical bone measures included bone area and total area (B.Ar and T.Ar; *i*.*e*., the mean cross-sectional areas of the analyzed volume), cortical thickness (Ct.Th), and cortical bone vBMD which describe the amount of bone contained in the region that was evaluated. Also included are cortical porosity (Ct.Po), which indicates subcortical resorption and is a contributor to bone strength, and maximum and minimum moments of inertia (I_max_ and I_min_), which contribute to bone structural integrity and strength.

Biomechanical properties of the femur were evaluated using three-point bending as previously described [34]. Briefly, femur length was measured via calipers (Mitutoyo, Kanagawa, Japan). Mechanical testing was performed in a 37 °C saline bath (MTS model 1/S, 100 N load cell; analyzed using TestWorks 4 software; MTS Corp., Minneapolis, MN) with testing support span width of 8 mm and crosshead speed of 0.17 mm/s.

### Gene expression studies

Total RNA was isolated from muscle tissue using mirVana kit from Invitrogen. Briefly, tissues were homogenized in kit lysis buffer with Matrix D beads in Fast Prep-24 Instrument for two 60 second runs at Speed 6. The homogenate was processed according to kit instructions and components using phenol:chloroform extraction and spin column purification. RNA integrity number (RIN) and concentration was determined using Nanodrop and Agilent Bioanalyzer using Agilent Nano kit. Total RNA samples with a RIN value below 6.0 were not processed further. The cDNA for each RNA sample was prepared using the High Capacity cDNA Reverse Transcription Kit (Applied Biosystems) according to kit instructions. For quadriceps, gastrocnemius, and tibialis anterior, cDNA reactions contained 5 μg total RNA per 100 μl, while for soleus, cDNA reactions contained 2 μg total RNA per 40 μl reaction. The cDNA was diluted 1:10 in water for quantitative PCR analysis. QPCR was performed with two technical replicates using TaqMan Fast Advanced Master Mix (Applied Biosystems), validated gene expression assays (Invitrogen), and eukaryotic 18s rRNA (Applied Biosystems) on the QuantStudio 7 Flex Instrument. Results are based on DeltaDelta CT calculations from gene of interest CT and 18s CT values.

### Statistical analyses

Measures collected at multiple time points during the study including grip strength and those from DEXA (lean mass and BMD) were evaluated by a repeated measures ANOVA, and confirmed using MANOVA to account for variations in sphericity, while considering factors of spaceflight, treatment, and time (0, 4, and 6 weeks post launch). Endpoint measures were analyzed statistically using Student’s t-test to compare baseline to controls, where appropriate, and using two-way Analysis of Variance (ANOVA) tested for group-wise comparisons for effects of spaceflight (ground vs flight) and treatment (IgG and YN41). Post-hoc analyses were performed using Dunnett’s test (micro-computed tomography) or Student’s paired *t* test. Statistical outliers were removed from QPCR data (defined as those beyonds 1.5 x Interquartile range from the first or third quartile) but not from other datasets. For all tests, a *P* value of < 0.05 was considered statistically significant. Error bars on all graphs show standard error (s.e.m.) from the mean.

## Results

Visual inspection of mice upon transfer and inspection of video footage of the mice in their on-orbit habitats after transfer, and daily throughout the mission, revealed that the mice adapted well to their habitats and appeared healthy. All flight and ground control mice survived the live phase portion of the experiment and appeared healthy at termination.

### Effects of myostatin inhibition on muscle function under conditions of microgravity

The results of the grip strength measures at interim and final timepoints showed significant main effects of both spaceflight and myostatin inhibition (p < 0.0001) and that grip strength was influenced by time following launch (p < 0.001). Statistically significant interactions were observed between time and spaceflight and time and myostatin treatment. Group-wise comparisons showed that grip strength in the Ground IgG control levels differed from baseline levels by -3.7% and +3.1%, respectively, at 4 and 6 weeks, respectively (Fig 1A). In comparison, administration of the myostatin antibody YN41 significantly increased grip strength by +14.6% over 4 weeks and +12.9% over 6 weeks. The increased grip strength results are consistent with previous reports using this antibody [26]. Muscle function significantly declined from baseline levels in Flight IgG group (-19.3% at week 4 and -7.8% at week 6), and at both timepoints the Flight IgG grip strength measurements were significantly lower than Ground IgG levels (p<0.05) at equivalent timepoints. These results demonstrated that microgravity conditions resulted in a decrease in muscle function as early as 4 weeks into flight. Grip strength increased at week 6 compared to week 4 in both flight animals and ground IgG controls, which might follow growth and maturation from the start of the flight experiment at 12 weeks of age. YN41 treatment of flight animals prevented the microgravity induced loss in muscle function. Indeed, the percentage change in grip strength from week 0 to 6-week of Flight YN41 animals was not significantly different to that of Ground IgG animals. Moreover, the percentage change in grip strength for YN41 Flight animals of 0.6% versus -7.8% for Flight IgG animals from week 0 to week 6 was similar in magnitude to the effect seen in the Ground YN41 group compared to its IgG group (12.9% vs 3.1%).

### Effects of microgravity and myostatin inhibition on skeletal muscle mass and body weight

To explore further the underlying physiology behind the effects of microgravity and myostatin inhibition on muscle function, *in vivo* body composition was analyzed using a DEXA densitometer at baseline, interim, and terminal timepoints. The results of this analysis are focused on lean mass in the hindlimb region (Fig 1B and 1C). Lean mass in ground control animals increased over the course of the experiment, likely due to normal growth during the study period, with main effects of both spaceflight (p = 0.066) and myostatin inhibition (p < 0.05). Lean mass changed with time following launch (p < 0.0001), and time was interactive with both spaceflight (p < 0.01) and myostatin inhibition (p < 0.01). Lean mass gains in Flight IgG animals were less than in ground IgG animals over the 6 week experiment (1.0 vs 3.5% gain at week 4, for Flight and Ground IgG groups, respectively; and 3.9% vs 5.6% gain at week 6), although the effect was not significant. In ground animals, YN41 treatment significantly increased lean mass at both 4 and 6 weeks as compared to untreated controls, and lean mass was also increased for treated flight animals as compared to untreated flight controls (3.6% vs 1.0% at week 4, for YN41 and IgG flight mice, respectively; and 7.9% vs 3.9% at week 6).

To determine if the observed changes in lean mass influenced body mass, the carcass weights (minus skin and the right leg) of all animals were assessed. The overall body weight in IgG control groups tended to decrease with microgravity exposure but the effect was not significant (Fig 1D). YN41-treated animals, however, were significantly heavier than their IgG controls, both in flight and on the ground, which was consistent with increases in lean mass observed using DEXA.

**Fig 2:**
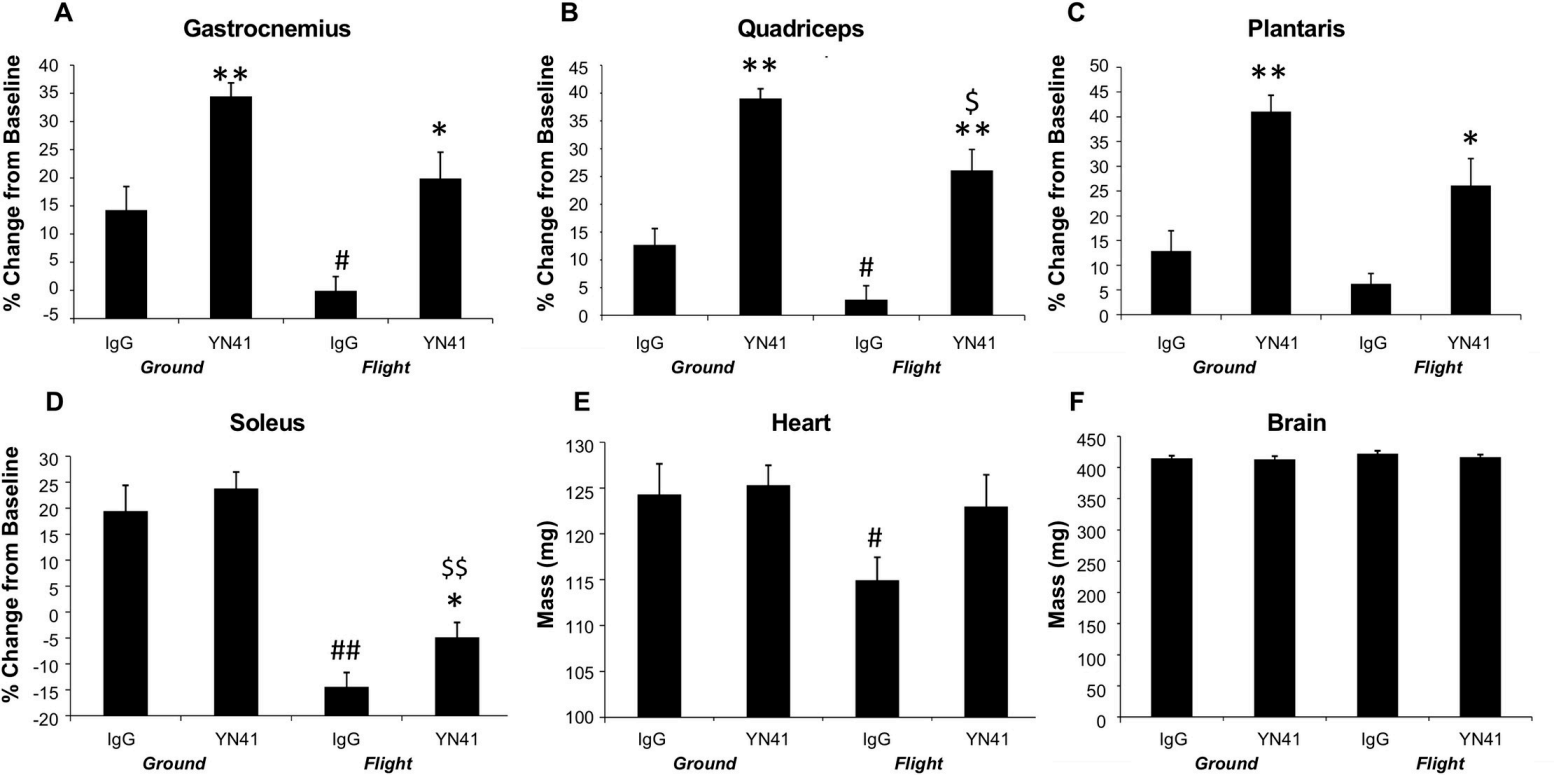
Effect of myostatin inhibition on muscle, brain and heart weights under conditions of microgravity. Individual weights of (A) gastrocnemius, (B) quadriceps, (C) plantaris, (D) soleus, (E) heart, (F) brain for Ground IgG, Ground YN41, Flight IgG and Flight YN41 groups at weeks 4 and 6 post-launch expressed as % change from the baseline group (A-D) or absolute weight (E-F). *, ** Indicates significance vs. its respective IgG control; #, ## indicates significance vs. ground IgG. p < 0.05, p<0.001.

Individual muscles were dissected and weighed. All muscles evaluated from the ground IgG group increased in weight (ranging from 7.5–19.5% over baseline values) over the 6-week course of the live phase experiment, and as expected of 12-week old growing mice (Table 1). In contrast, both fore-limb and hindlimb muscles (except for the EDL) from Flight IgG mice did not gain as much as the ground controls over the course of the experiment. The flight gastrocnemius did not gain any weight compared to the baseline group (Fig 2A) and the flight soleus decreased by -14.4% as compared to baseline values (Fig 2D). The quadriceps (Fig 2B) and tibialis anterior (TA) muscles from Flight IgG mice were also significantly lighter compared to those from Ground IgG mice. Comparably, the EDL appeared not to atrophy from spaceflight, in line with muscle masses [13] and gene expression changes observed in prior studies of spaceflight flown mice [13]. Two-way ANOVA showed a main effect of microgravity exposure affected all but the triceps brachii and EDL muscles. Overall, our results support the reduced hindlimb lean mass and muscle strength observed in Flight IgG animals as compared to the ground IgG group.

**Table 1:** Effect of myostatin inhibition on individual muscle weights under conditions of microgravity.

Muscle	Weight										
	Baseline	Ground IgG		Ground YN41		Flight IgG		Flight YN41			
	mg	mg	% Change from Baseline	mg	% Change from Ground IgG	mg	% Change from Ground IgG	mg	% Change from Ground IgG	% Change from Flight IgG	2 way ANOVA *MI*, *SF*, *Int*
Triceps brachii	73.3±1.8	81.9±2.2	11.8±3.0*	107.8±2.3	31.6±2.7**	78.3±3.9	-4.4±4.8	100.3±2.5	22.4±3.1**	28.0±3.4**	*‡‡‡*, *†*, *NS*
Gastrocnemius	83.6±1.7	95.5±3.3	14.3±4.0*	112.4±2.0	17.7±2.0**	83.6±2.0	-12.5±2.1*	100.3±3.7	4.9±3.9	20±4.4*	*‡‡‡*, *‡‡‡*, *‡‡*
Quadriceps	141.0±4.9	158.8±3.9	12.7±2.8*	196.0±2.5	23.4±1.5**	144.9±3.4	-8.8±2.1*	177.7±5.1	11.9±3.2*	22.6±3.5**	*‡‡‡*, *‡‡*, *NS*
Tibialis anterior (TA)	44.1±1.6	51.6±1.1	16.9±2.6*	58.2±1.8	12.9±3.3*	46.7±1.1	-9.5±2.1*	59.2±0.8	14.8±1.5**	26.9±1.7**	*‡‡‡*, *‡‡‡*, *††*
Extensor digitalis longissimus (EDL)	8.3±0.2	8.9±0.2	7.5±2.6	10.8±0.3	21.8±3.7**	9.0±0.3	1.5±3.6	10.0±0.3	12.2±3.4*	10.5±3.3	*‡‡‡*, *NS*, *NS*
Plantaris	10.1±0.4	11.4±0.4	12.9±3.8*	14.2±0.3	24.9±2.8**	10.7±0.2	-5.9±1.7	12.7±0.5	11.7±4.6	18.7±4.9*	*‡‡‡*, *‡*, *NS*
Soleus	5.6±0.2	6.6±0.3	19.5±4.7*	6.9±0.2	3.6±2.5	4.8±0.2	-28.4±2.2**	5.3±0.2	-20.4±2.3**	11.2±3.2*	*†*, *‡‡‡*, *NS*

Two-way ANOVA evaluated for main effects of myostatin inhibitor treatment (MI: IgG and YN41) and spaceflight (SF: ground vs flight), and interactions (Int) between SF and Tmt; p-values are reported for † = p < 0.10, †† = p < 0.05, ‡ p < 0.01, ‡‡ p < 0.001, ‡‡‡ p < 0.0001, and NS indicates no significance. For % change over Baseline, Ground IgG or Flight IgG comparisons, ^*^ indicates significance p < 0.05, ^**^ indicates significance p < 0.001; data are presented as mean ± s.e.m.

Myostatin antibody treatment of ground control mice significantly increased the weight of 6 of the 7 individual muscles isolated (Table 1, Fig 2A–2C) from 12.9–31.6% depending on the individual muscle. The soleus, a type I slow oxidative muscle, showed an increased in weight with YN41 treatment, but the effect was not significant (Fig 2D). All other muscles (except the EDL) isolated from Flight YN41 mice were significantly heavier than the Flight IgG controls (Fig 2A–2C), ranging from 10.5–28.0% heavier depending on the individual muscle, and demonstrating that myostatin inhibition can occur under conditions of microgravity. Notably, 5 of these muscles were also significantly heavier than their Ground IgG counterparts demonstrating that myostatin inhibition was able to prevent all the muscle atrophy induced by conditions of microgravity. It is interesting to compare the degree of effect of myostatin inhibition on individual muscles on Earth as compared to on the ISS. A number of the muscles such as the gastrocnemius, quadriceps and triceps responded equally well in the presence or absence of gravity. The TA and soleus appeared to respond better under microgravity conditions as compared to equivalent ground controls, whereas the EDL and plantaris responded to a lesser degree than their ground control muscles (Table 1).

Heart and brain weights were collected from all five treatment groups (Fig 2E and 2F, respectively). Heart weights were significantly lighter for the Flight IgG group compared to the Ground IgG group; two-way ANOVA also showed a main effect of spaceflight but not treatment for heart mass. YN41 treatment overcame this reduction. YN41 treatment did not affect heart weights of ground controls. Brain weights did not change over the course of the live phase of the experiment and neither microgravity and/or myostatin inhibition affected this outcome.

### Effect of microgravity and myostatin inhibition on muscle histomorphology and fiber size

**Fig 3:**
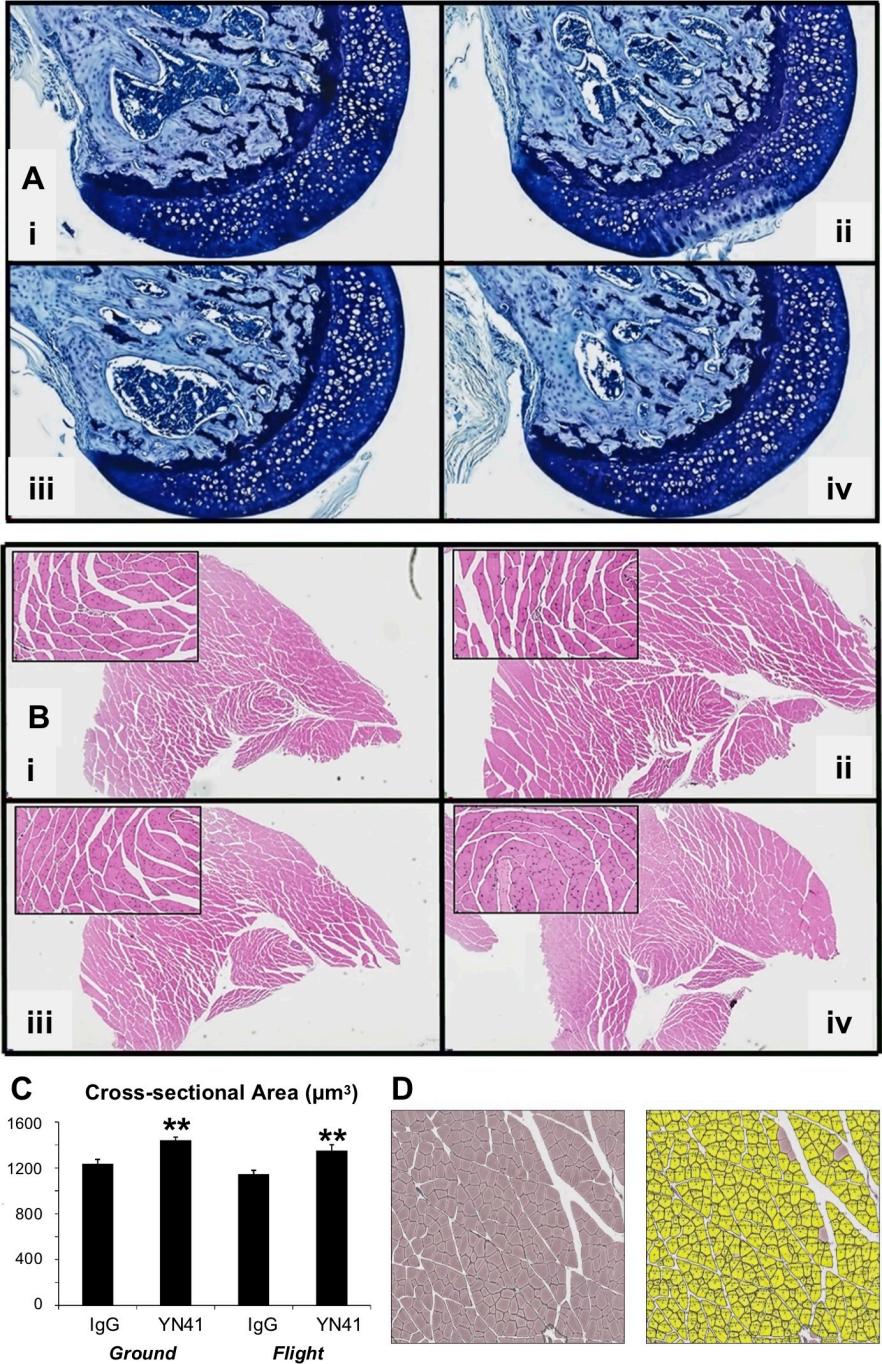
Effect of myostatin inhibition and microgravity on femoral head and muscle histology and myofiber size. A). No degenerative or inflammatory changes were microscopically observed in observed in longitudinal sections of the femoral head near the ligament of the head of the femur (A) or in transverse sections of the lower hind limb muscles (B) in control or YN41 treated mice housed on the ground or in flight. i) Ground IgG, ii) Ground YN41, iii) Flight IgG, iv) Flight YN41. High magnification inset in B is of the soleus muscle (centrally located). (C). Mean cross-sectional areas of lower limb hindlimb muscle fibers from ground and flight groups, where two-way ANOVA showed main effects of both myostatin inhibition (p < 0.0001) and spaceflight (p < 0.05). **p<0.001 vs their respective IgG controls. D). Representative image of a lower limb muscle section (top) stained to highlight muscle fibers and its corresponding image analysis mask (bottom) used as part of the image analysis process.

To explore the effects of microgravity and myostatin inhibition on underlying hindlimb morphology, sections of hindlimb muscle bundles and femoral heads from flight and ground control mice were examined histologically. It has been suggested that cartilage integrity may be compromised by long-term spaceflight [35]. To this end, femoral heads from mice exposed to microgravity were examined but showed no inflammatory or degenerative changes regardless of drug treatment (Fig 3Ai-iv). Muscles from ground and flight mice without YN41 treatment also appeared histologically normal (Fig 3Bi-iv).

Since microgravity had been shown to reduce individual muscle masses in the hindlimbs of flight mice, it was of interest to determine if this was due to an atrophy of existing myofibers. Myofiber size of muscles in the hindlimb lower muscle bundle was assessed using an image analysis algorithm, where image analysis tools recognize and outline myofibers for measurement of cross-sectional area (Fig 3D). Although there was a decrease in CSA in muscles from Flight IgG compared to Ground IgG (Fig 3C), the decrease was not significant. YN41 treatment, however, caused a hypertrophy of myofibers for both control and flight mice as shown by the significant increase in myofiber CSA.

### Effect of microgravity and myostatin inhibition on muscle gene expression

**Fig 4:**
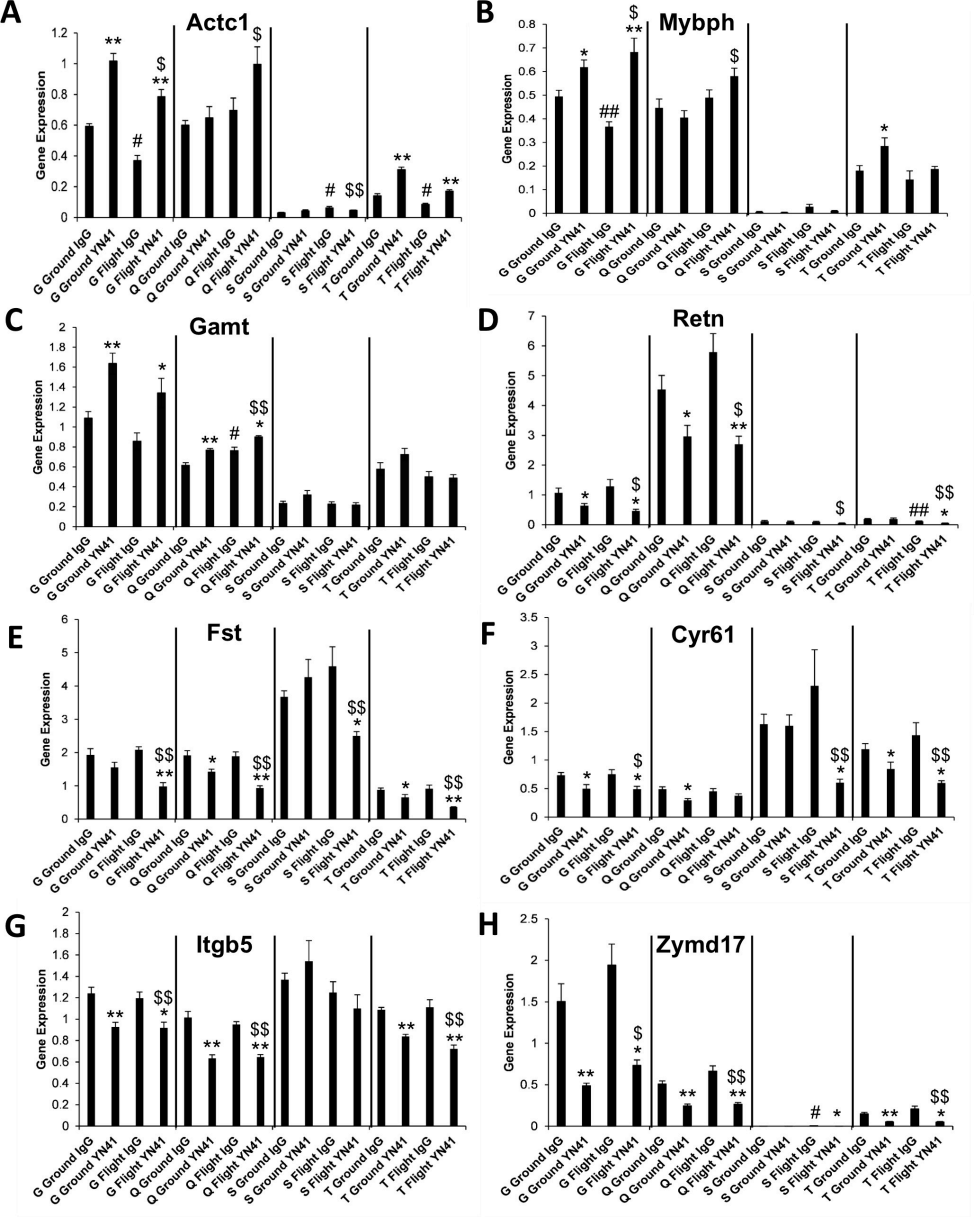
Effect of microgravity and myostatin inhibition on skeletal muscle gene expression. Gene expression profiles of 8 genes in gastrocnemius (G), quadriceps (Q), soleus (S) and tibialis anterior (T) muscles of flight and ground animals. Significance at p<0.05, p<0.001 relative to the respective control groups (*,**), to the Flight IgG group (#,##) and to the Ground IgG group ($,$ $).

To investigate the molecular effects of microgravity and myostatin inhibition on skeletal muscle of ground and flight mice, gene expression was analyzed using QPCR. Three classes of genes were selected for analysis: i) genes previously described to be modulated by microgravity, ii) genes belonging to the myostatin pathway, iii) genes known to be modulated by myostatin inhibition (previously published or from our own unpublished observations). Some genes fit into more than one category. The genes, their descriptions and the primer probe kits used for these analyses are listed in S1 Table. The expression results for these 28 genes in the gastrocnemius, quadriceps, soleus and tibialis anterior muscles are tabulated in S2 Table. It is to be noted that these genes often had very different levels of expression between the 4 muscles examined in control ground-dwelling mice. For example, Mybph, Actc and Resn were expressed more highly in the fast-twitch quadriceps muscle but virtually undetectable in the slow-twitch soleus. Conversely, Fst and Cyr61 and Pax7 were expressed at a higher level in the soleus than the quadriceps. Even within fast twitch muscles, gene expression levels varied in untreated ground control mice. For example, Cidec1 was highly expressed in the quadriceps but at 10-fold less levels in the gastrocnemius and TA. The reasons for these varying gene expression levels between individual muscles is unclear. It was of interest to determine if microgravity had modulated gene expression in skeletal muscle concomitant with inducing reductions in muscle mass. Alpha cardiac actin (Actc1) was significantly affected by microgravity in 3 out of the 4 muscles examined (Fig 4A). A decrease in gene expression in Flight IgG animals compared to Ground IgG animals was seen in the gastrocnemius and TA muscles whereas an increase was seen in the soleus. Guanidinoacetate methyltransferase (Gamt), an N-methyltransferase that catalyzes synthesis of creatine, was significantly increased by spaceflight in the quadriceps (Fig 4C), whereas Myosin binding protein H (Mybph), was significantly decreased by microgravity in the gastrocnemius (Fig 4B). A decrease in expression of resistin (Retn) in the TA in response to spaceflight was observed (Fig 4D). The next set of 17 genes examined had previously been described as being modulated by spaceflight [11,13,36]. A significant decrease in Frzd9 expression in the gastrocnemius in flight animals was observed, reproducing the result of Allen et al. [36], with an increase seen in the TA (S2 Table). Kcnma1 was significantly decreased (-1.72 fold) in the soleus in flight animals in accordance with the result of Gambara, et al. [11]. Trim63 (MurF1) showed a significant increase in the soleus, in accordance with data from Sandona, et al. [13]. Pitx2, a gene shown to be increased by microgravity in the gastrocnemius [36], showed a trend for an increase in flight IgG animals compared to ground controls and a significant increase (+1.54 fold) in flight animals in the quadriceps. Ppargc1a showed a significant decrease in flight in the gastrocnemius but did not reproduce the previously decrease in flight in the soleus [11]. Rather than a decrease seen in flight in the gastrocnemius of Slc38a2 expression as reported previously [36], a significant increase in this gene in flight animals was observed (S2 Table). No significant changes were seen in muscles from flight IgG mice compared to ground IgG mice in the expression of Cfd, Cidec, Cyr61 (Fig 4F), Dnajb1, Fasn, Foxo1, Fst (Fig 4E), Fstl1, Id1, Myf6 or Rbp4 (S2 Table), all genes previously reported to be modulated by microgravity.

A number of significant changes in gene expression were induced by myostatin inhibition, particularly in the fast twitch muscles (i.e., gastrocnemius, quadriceps and tibialis anterior) on which YN41 had a pronounced effect on muscle mass. Effects on gene expression were less pronounced in the soleus, in keeping with the lesser effect of myostatin inhibition on the weight of this muscle. In general, effects of myostatin inhibition on gene expression were similar in flight as in ground animals. Actc1 and Mybph both showed increases in expression in the gastrocnemius and TA in both ground and flight animals with YN41 treatment (Fig 4A and 4B). Previously Latres et al. [37] described that treatment of mice with a myostatin antibody increased expression of Actc1 and Mybph in the TA. Interestingly, for both Actc1 and Mybph, myostatin inhibition modulated expression in the opposite direction to the effect of microgravity. Notably, myostatin inhibition in the gastrocnemius was able to overcome the effects of microgravity and restore gene expression levels to at least those of Ground IgG controls (Fig 4A). Gamt was also significantly increased in the gastrocnemius and quadriceps of both ground and flight animals with myostatin inhibition although in the quadriceps microgravity also increased Gamt expression. For Trim63, myostatin inhibition in the soleus of flight animals was shown to significantly decrease expression, whereas microgravity modulated the gene in the opposing direction (S2 Table). A number of genes eg Fst, Cyr61, Itgb5, Resn and Zymd17 were significantly decreased by YN41 treatment in both Ground and Flight animals and across multiple muscles (Fig 4D–4H). Others have previously reported a decrease of Zymd17 in the TA of myostatin antibody-treated mice [37]. The only effect of microgravity on these genes was in a decrease in the TA of Resn in flight animals and an increase in the soleus of Zymd17. However, since the expression of these 2 genes was very low in these specific muscles, the significance of this finding is unclear. Other genes that were influenced by YN41 treatment, either on the ground or in flight, in at least one muscle were Acrv2b, Dnajb1, Fasn, Fstl1, Frzd9, Igfbp5, Pax7, Pitx2, Ppargc1a and Rbp4 (S2 Table). Myostatin gene expression was shown to be increased by YN41 treatment in the gastrocnemius, perhaps as a compensatory mechanism for myostatin protein sequestration (S2 Table).

### Effect of microgravity and myostatin inhibition on bone

**Fig 5:**
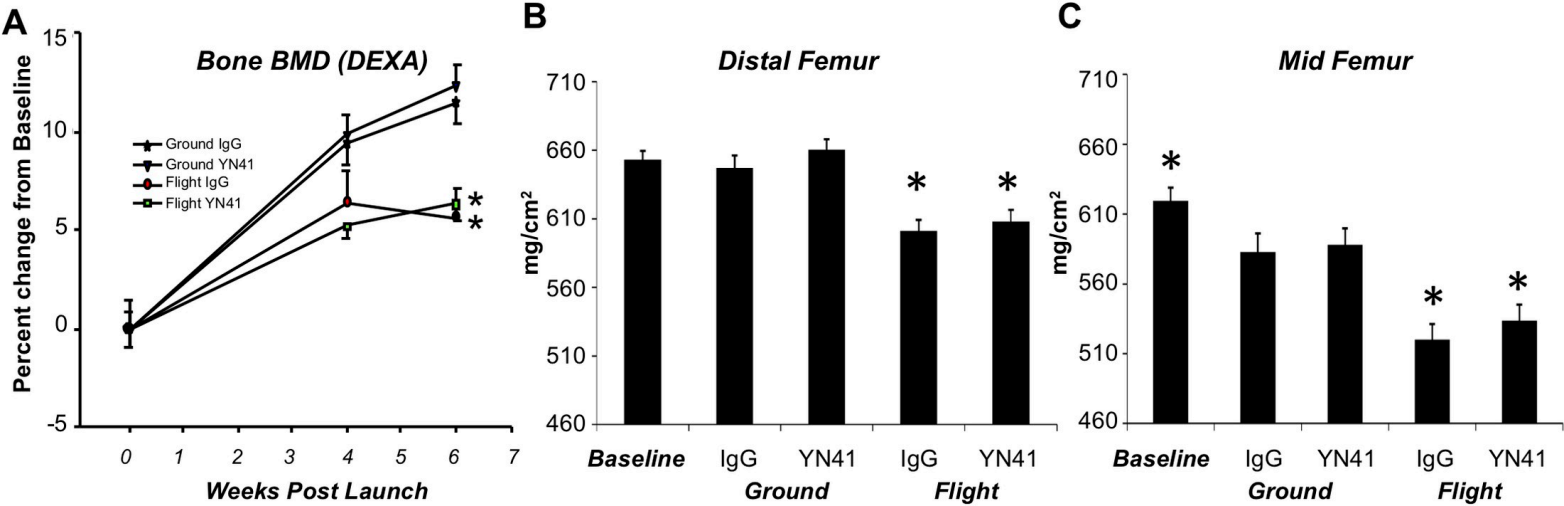
Effect of microgravity and myostatin inhibition on bone density. A. Areal bone mineral density measured *in vivo* by DEXA in Ground IgG, Ground YN41, Flight IgG and Flight YN41 groups at weeks 4 and 6 post-launch expressed as % change from the same groups measured at week 0. (*), refers to significance relative to its respective control at each time point. All p values < 0.05. BMD of distal (B) and mid (C) femurs as measured by *ex vivo* CT. * denotes significance to Ground IgG, p<0.05. Two-way ANOVA revealed significant main effects of spaceflight, but no significant effect of myostatin inhibition, for areal BMD at both 4 and 6 weeks post-launch (p < 0.01 and p < 0.0001, respectively) and BMD of distal and mid-femurs (p < 0.0001).

The effects of microgravity and myostatin inhibition on bone health were evaluated via DEXA (i.e., for bone mineral density), micro-computed tomography (e.g., for areal bone mineral density and microarchitectural assessment), and mechanical testing (e.g., for strength). Interim (4 and 6 weeks post launch) and terminal *in vivo* DEXA bone densitometer readings were taken for flight and ground mice and the data are shown in Fig 5A. Microgravity caused a significant decrease in areal bone mineral density (BMD) in the region of the hindlimb (region shown in Fig 1C) in Flight IgG animals compared to their Ground IgG counterparts (Fig 5A). The decrease in areal BMD in flight mice was apparent after 4 weeks onboard the ISS, where the group-wise effect of spaceflight was significant (p < 0.01). Myostatin inhibition had no significant effect on BMD from DEXA either on the ground or in flight; however, time following launch was highly significant (p < 0.0001) and time was interactive with spaceflight (p < 0.0001) but not myostatin inhibition treatment. To examine the effect on bone density in more detail, femurs and vertebrae were examined for bone-specific areal BMD by computed tomography (Table 2) and then for microarchitectural assessment at the distal femur. As seen with *in vivo* DEXA bone measures, *ex vivo* CT confirmed that conditions of microgravity resulted in a significant decrease in areal BMD in the femur but not in the vertebrae. Mid- and distal femoral areal BMD were -10.76% and -7.10% lower in Flight IgG animals compared to Ground IgGs, respectively (Table 2, Fig 5B and 5C). However, lumbar vertebral areal BMD was unaffected by conditions of microgravity (Table 2). As observed in BMD measurements from DEXA, myostatin inhibition had no significant effect on femoral or vertebral BMD as measured *ex vivo* (Table 2, Fig 5B and 5C).

**Table 2:** Age and microgravity, but not myostatin inhibition, influence vBMD in the femur but not the vertebrae. Age and microgravity, but not myostatin inhibition, also influence both trabecular and cortical bone microarchitecture at the distal femur.

	Baseline	Ground IgG	Ground YN41	Flight IgG	Flight YN41	2 way ANOVA *MI*, *SF*
***Bone-Specific Areal BMD Measures (all in mg/cm***^***2***^***)***						
Mid- Femur	619.6 ± 8.9 †	582.8 ± 12.8 ^A^	588.0 ± 11.2 ^A^	520.1 ± 10.4 ^B^	533.5 ± 11.0 ^B^	‡‡‡, N.S.
Distal Femur	653.1 ± 6.0	647.0 ± 8.7 ^A^	660.3 ± 7.3 ^A^	601.1 ± 7.8 ^B^	608.1 ± 7.9 ^B^	‡‡‡, N.S.
Lumbar Vertebrae	324.9 ± 9.4	343.5 ± 10.9	363.3 ± 10.8	356.3 ± 12.7	356.6 ± 13.0	N.S., N.S.
***Trabecular Bone Microarchitecture Measures***						
Bone Volume Fraction, BV/TV (%)	12.4 ± 0.9	14.6 ± 1.0	13. 6 ± 1.0	11.2 ± 1.2	12.1 ± 0.5	‡, N.S.
Trabecular Number, Tb.N (1/μm)	3.19 ± 0.17	3.61 ± 0.17	3.42 ± 0.16	2.97 ± 0.22	3.19 ± 0.10	††, N.S.
Trabecular Thickness, Tb.Th (μm)	37.3 ± 1.0	39.5 ± 1.2	38.8 ± 1.1	36.0 ± 1.3	37.1 ± 0.8	††, N.S.
Trabecular Spacing, Tb.Sp (μm)	286 ± 21	245 ± 17 ^A^	260 ± 15 ^AB^	319 ± 29 ^B^	279 ± 9 ^AB^	††, N.S.
Tb.vBMD	153.5 ± 9.0	183.1 ± 10.9 ^A^	169.5 ± 11.0 ^AB^	140.6 ± 13.0 ^B^	152.4 ± 6.3 ^AB^	‡, N.S.
***Cortical Bone Microarchitecture Measures***						
Bone Area, BA (mm^2^)	0.89 ± 0.01 †	0.94 ± 0.02 ^AB^	0.99 ± 0.02 ^A^	0.88 ± 0.02 ^B^	0.88 ± 0.02 ^B^	‡‡‡, N.S.
Total Area, TA (mm^2^)	1.09 ± 0.01	1.11 ± 0.02 ^AB^	1.17 ± 0.02 ^A^	1.06 ± 0.02 ^B^	1.05 ± 0.03 ^B^	‡‡, N.S.
Cortical Thickness, Ct.Th (μm)	134.35 ± 1.85 ‡	151.10 ± 1.84 ^AB^	155.82 ± 1.67 ^A^	148.96 ± 2.02 ^B^	147.26 ± 1.61 ^B^	‡, N.S.
Cortical Porosity, Ct.Po (%)	18.1 ± 0.5 ‡	15.3 ± 0.3 ^AB^	14.9 ± 0.4 ^B^	16.5 ± 0.5 ^A^	15.9 ± 0.4 ^AB^	‡, N.S.
I_max_ (mm^4^)	0.43 ± 0.01 §	0.48 ± 0.01 ^AB^	0.52 ± 0.01 ^A^	0.45 ± 0.01 ^B^	0.44 ± 0.02 ^B^	‡‡, N.S.
I_min_ (mm^4^)	0.20 ± 0.00 §	0.22 ± 0.01	0.23 ± 0.01	0.21 ± 0.00	0.21 ± 0.01	††, N.S.
Ct.vBMD (mg HA/ccm)	892.0 ± 5.5 ‡	944.6 ± 2.9	948.8 ± 8.7	935.3 ± 5.2	929.8 ± 6.8	††, N.S.

Two-way ANOVA evaluated for main effects of myostatin inhibitor treatment (MI: IgG and YN41) and spaceflight (SF: ground vs flight); no interactions were observed. P-values are reported for †† = p < 0.05, ‡ p < 0.01, ‡‡ p < 0.001, ‡‡‡ p < 0.0001, and NS indicates no significance.

Data are reported as mean ± s.e.m. Baselines were compared to ground IgG groups using a Student’s t-test to evaluate for influence of age during the flight experiment. All differences are noted as † for p < 0.05 § for p < 0.01, ‡ for p < 0.001. Two-way ANOVA evaluated for effects of spaceflight (i.e., Ground vs. flight) and treatment (i.e., IgG vs. YN41), where a Dunnett’s post-hoc test compared each treatment group to IgG Ground Control (where groups not connected by the same letter are significantly different); α = 0.05.

**Fig 6:**
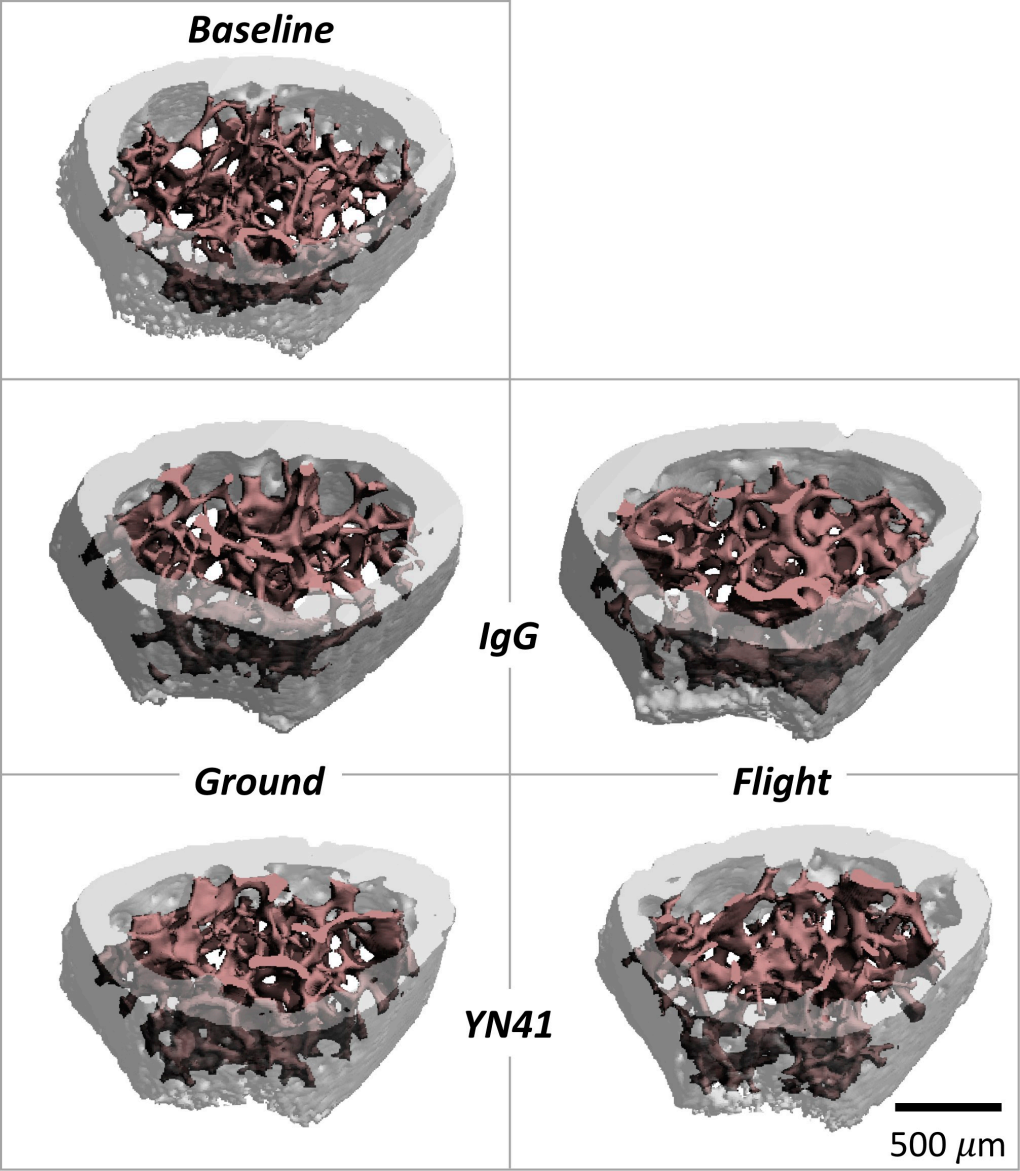
Representative micro-computed tomography images from the distal femur from baselines as well as ground and flight mice treated with either IgG or YN41. While visual differences between groups are subtle, the microarchitecture of both trabecular and cortical compartments significantly improved with skeletal maturation when comparing baseline to ground controls (IgG), and both bone compartments were significantly diminished with microgravity exposure (comparing IgG ground to flight groups) but were unaffected by myostatin inhibition.

Microarchitectural assessment of trabecular and cortical bone at the distal femur demonstrated significant bone deterioration with microgravity exposure with no effect of myostatin inhibition (Table 2; Fig 6). IgG ground control mice demonstrated skeletal growth, via increased bone geometric measures, as compared to their younger, Baseline counterparts primarily in cortical, and not trabecular, bone with significant increases in BA, Ct.Th, maximum and minimum cross-sectional moments of inertia, and volumetric bone mineral density in cortical bone, along with significantly decreased cortical porosity. In addition, both cortical and trabecular bone were affected by unloading in microgravity with no significant effect of myostatin inhibition. Comparisons showed a consistent pattern of diminished cortical and trabecular bone microarchitecture across all standard μCT measures following microgravity exposure (Table 2).

Finally, three-point flexural testing was used to assess the influence of microgravity-induced bone decrements on mechanical properties (Table 3). Despite the decrease in BMD seen in the femurs of flight mice, stiffness, energy to break, peak load and ultimate elongation parameters were not significantly affected compared to their ground counterparts. Myostatin inhibition had no effect on biomechanical properties (Table 3).

**Table 3:** Effect of microgravity and myostatin inhibition on femoral biomechanics.

Femoral Biomechanics				
Group	Stiffness (kgf/mm)	Peak Load (kgf)	Energy to Break (kgf x mm/mm^3^)	Ultimate Elongation (mm)
Baseline	8.7±0.45	1.7±0.07	0.28±0.01	0.27±0.01
Ground IgG	9.9±0.45	1.9±0.06	0.30±0.02	0.27±0.01
Ground YN41	9.9±0.25	1.9±0.04	0.35±0.02	0.29±0.01
Flight IgG	9.4±0.18	1.8±0.04	0.30±0.02	0.27±0.01
Flight YN41	9.7±0.43	1.9±0.05	0.29±0.01	0.26±0.01

Data are shown ± s.e.m.

## Discussion

Rodent Research-3, sponsored by the ISS National Laboratory, launched to the ISS aboard NASA's eighth cargo resupply flight of the SpaceX Dragon spacecraft (SpX-8) on April 8, 2016. The primary goal of the RR-3 investigation was to determine if inhibition of myostatin, through delivery of a neutralizing anti-myostatin antibody, could prevent the loss of skeletal muscle mass that was expected to occur in mice under conditions of microgravity. The live phase of the experiment was one of the longest to date, being 6 weeks in length, and involved female BALB/C mice that were 12 weeks at the time of launch. Both interim and terminal measures were collected, made possible by new onboard anesthesia recovery hardware and procedures. The mission was also unique in that it measured muscle function with a grip strength meter during flight, and so for the first time could evaluate longitudinal functional consequences of microgravity disuse in addition to endpoint changes in muscle mass.

Microgravity conditions resulted in overall loss of muscle mass and underlying bone in the flight mice compared to ground controls. Total carcass weight was also decreased with spaceflight, although not significantly. Lean mass loss was apparent by 4 weeks as evidenced by interim DEXA measures and progressed through 6 weeks of microgravity exposure. Of the 7 individual muscles collected at termination, the soleus muscle atrophied the most, losing 28.4% of weight compared to the ground controls and falling below baseline levels. Others have reported a similar dramatic atrophy of this type I slow-twitch postural muscle during spaceflight as measured by reduced myofiber CSA [8,11,13]. The predominantly fast-twitch type II gastrocnemius muscle also showed significant loss (-12.5%) in mass compared to ground controls consistent with previous histological findings [8]. The EDL showed no evidence of wasting, consistent with the reported maintenance of gene expression [11] and myofiber CSA [13] following spaceflight. Similarly, EDL mass was unchanged in hindlimb suspended mice in 1g [38]. In contrast, grip strength measurements demonstrated that muscle strength in flight animals was decreased by 15.6% more than ground controls at 4 weeks into flight. This is the first direct evidence of muscle weakness in mice as measured during spaceflight. Microgravity also affected underlying bone. *In vivo* DEXA showed a decrease in hindlimb BMD and *ex vivo* measures confirmed a decrease in distal and mid-femur BMD. Microarchitectural assessment of cortical and trabecular bone at the distal femur showed consistent detrimental influences of microgravity exposure, yet no influence of the myostatin inhibition. Despite these decreases, no significant effects on tibial biomechanical properties were seen in flight animals compared to their ground counterparts. Lumbar vertebral areal BMD was not affected by spaceflight. Mice were not skeletally mature throughout the duration of this study [39–41], where skeletal maturity is generally considered to occur around 20 weeks of age. Therefore it is possible that ongoing bone formation was sufficient to mitigate the catabolic effects of microgravity exposure in these growing, female BALB/c mice over the 6-week duration of the live phase study. However, other studies in similarly aged female BALB/c and C57BL/6 mice reveal significant losses after two-weeks of microgravity exposure [12,18]. These results suggest the need for future studies to evaluate the effects of age, duration of microgravity exposure, and how genetic background may differently affect bone in spaceflight.

Inhibiting the growth factor myostatin with a neutralizing antibody has been shown in a recent proof-of-concept clinical trial to be able to increase lean mass and power measures in an elderly weak population [27]. The humanized myostatin antibody used in that trial was derived from the murine antibody used in this study (YN41, LSN2478185). As shown in earlier work with rodent models [26], myostatin inhibition was able to increase body weight, lean mass, individual muscle weights and muscle strength in mice under regular ground conditions. The effect of myostatin inhibition in this study was significant on predominantly fast-twitch muscles (ranging from an increase of 31.6% in weight for the triceps to 12.9% for the TA), with a trend for an increase of 3.6% increase for the slow-twitch type I soleus muscle. This is consistent with previous reports of myostatin inhibition [26]. Notably, myostatin and ActRIIb mRNA levels are greater in fast- than in slow-twitch muscles [42]. In this study, myostatin inhibition was able to increase lean mass, muscle weights and muscle strength in flight animals compared to the IgG flight controls in an overall manner similar to the effect seen on ground control animals, demonstrating that muscle building effects of myostatin inhibition including increases in function do not require the weight-bearing effects of gravity. In fact, the myostatin antibody was able to prevent all of the losses in hindlimb lean mass, grip strength and muscle weights (with the exception of the soleus) induced by microgravity. For some individual muscles *e*.*g*., quadriceps, triceps, TA, plantaris, myostatin inhibition significantly increased weight over that of ground IgG controls, demonstrating that YN41 was not only able to overcome the muscle atrophy induced by spaceflight but induce even larger effects. Mice in the NASA flight hardware modules exhibit species-typical behaviors including running (i.e., circularly about the cage) and grasping the wire mesh walls, the ground control mice are also active [43]. The triceps brachii muscles may provide insight into relative activity differences between flight and ground mice; however, these masses do not explain the increased grip strength of mice treated with the antibody (e.g., regression of grip strength change by 6 weeks poorly correlates with triceps brachii masses, R^2^ = 0.15). However, myostatin antibody resulted in myofiber hypertrophy, as measured by myofiber CSA, as expected. As on the ground, effects of myostatin inhibition were larger on fast-twitch muscles than on the slow-twitch soleus muscle.

However, some differences in response to myostatin inhibition between flight and ground animals were noticeable. The increase in hind limb lean mass and muscle strength induced by myostatin inhibition was not as large in flight animals as compared to ground controls, supported by the plantaris (24.9% ground vs 18.7% flight). However, in contrast, the weights of the TA and soleus muscles were increased more in YN41-treated flight animals compared to YN41-treated ground controls (12.9% ground vs 26.9% flight for TA, 3.6% ground vs 11.2% flight, versus their respective IgG controls). The reason for these differences in responsiveness is unclear and needs to be confirmed.

At the molecular level microgravity was shown to result in decreases in RNA encoding muscle cytoskeletal proteins Actc1 and Mybph. This is consistent with the reduction in muscle mass that had occurred in the skeletal muscles. Trim63 (MurF1) expression increased in space consistent with the activation of the ubiquitin-proteosome pathway to induce muscle atrophy [44]. Changes in Frzd9 and Kcnma1 gene expression induced by spaceflight appear robust since these changes reproduced those previously reported [11,36]. Lack of reproducibility of changes in expression of other genes reported by others could be due to differences in spaceflight duration or mouse strain. Not surprisingly, many of the directional changes in gene expression induced by spaceflight seen in type II muscles (i.e., gastrocnemius, quadriceps, TA) were not seen in the type I soleus muscle (e.g., Actc1) and vice-a-versa (e.g., Acrv2b and Alk4). Regarding the effects of myostatin inhibition on gene expression, the changes observed in the 28 genes examined were more plentiful than microgravity, perhaps not surprising since the effect of YN41 treatment on muscle weights was larger–at least for fast twitch muscles. Myostatin inhibition does ‘reverse’ some of the effects of microgravity on underlying muscle gene expression, notably Actc1 and Mybph. Spaceflight reduces expression of these genes and YN41 treatment increased their expression. As these are genes that encode proteins involved in muscle architecture, it might be expected that microgravity should reduce expression and myostatin inhibition should increase. However, more often it appears that myostatin inhibition modulates genes that are not affected by microgravity and vice-a-versa. For example, Itgb5, Fst and Cyr61gene expression was consistently reduced by YN41 but these genes were not affected by spaceflight. It is of interest to note that Cyr61 is increased in human skeletal muscle after mechanical loading [45]. The decrease in Fst induced by myostatin inhibition is perhaps an attempt to compensate for the decreased levels of active myostatin by reducing levels of one of its natural inhibitors. The increase in expression of Gamt both in YN41-treated flight and ground mice is interesting and may contribute to the increase grip strength measured in these groups as Gamt deficient mice have been shown to have lower force than their normal counterparts [46,47]. The decrease in Retn [46] with YN41 treatment in both flight and ground groups is novel and the decrease in resistin levels with increased muscle strength due to resistance training in the elderly is of note.

Myostatin inhibition was not able to reverse bone loss that was observed in flight animals, despite the dramatic increase induced in muscle mass with treatment. Here, spaceflight resulted in diminished bone mass and microarchitecture, yet myostatin inhibition did not show any significant effect on BMD even with the weight-bearing effect of increased muscle mass in ground controls. This was not unexpected as myostatin inhibition did not show any significant effect on BMD even with the weight-bearing effect of increased muscle mass in ground controls and was consistent with previous observations [48]. While it is possible that increased muscle mass and strength may have had an effect on bone outcome measures, and in particular microCT assessment of the distal long bones, we observed no such relationships in the present study or in a prior study of female C57BL/6 mice that were flown on the Space Shuttle for ~two weeks (unpublished data). It is likely that the increased loading from muscles of greater mass and strength following myostatin inhibition caused small, but insignificant increases in bone mass. Site-specific assessment of bone microarchitecture and material properties (e.g., via nanomechanical testing) would be needed to evaluate for bone changes proximal to sites of muscle attachments. Also, a longer duration experiment could have allowed the more slowly adapting bone tissue adjust to increased skeletal muscle mass and strength.

The mice in flight, regardless of treatment group, adapted well to their new environment and appeared healthy through the 6 weeks of the experiment. However, the flight mice did exhibit ‘racetrack’ behavior by 2 weeks [43], which would likely provide physical activity and some degree of musculoskeletal loading during spaceflight. However, there was no detectable effect of this running behavior or gripping the wire mesh walls of the NASA habitats on the triceps brachii masses or grip strength. It is noteworthy that heart weight was reduced in the Flight IgG group compared to ground controls. Myostatin inhibition was able to prevent that loss in flight animals, although on the ground the myostatin antibody-treated group had no effect on heart weight. Myostatin inhibition has previously been shown to not affect heart weight.

In summary, mice in space are a useful model of muscle atrophy since effects are global and can be maintained for many weeks if not months, unlike many ground models of muscle wasting. Myostatin inhibition prevented the muscle atrophy and accompanying muscle weakness seen in mice that were flown aboard the ISS for six weeks, one of the longest rodent flights to date, thus demonstrating myostatin inhibition as an effective countermeasure to this detrimental consequence of life under microgravity conditions. A number of myostatin pathway inhibitors are in clinical trials for muscle atrophy and have been shown to increase muscle mass and power measures in man [27,49–51]. This raises the future possibility in astronauts and cosmonauts of therapeutic intervention to counteract muscle atrophy induced by long term spaceflight and may be important for successful missions to the Moon and Mars.

## Supporting information

S1 Table (DOCX)

S2 Table Gene expression values for gastronemius, quadriceps, soleus and tibialis anterior muscles from flight and ground groups. (DOCX)

S1 Raw Data (XLSX)
