# Supplementary material for: Inhibition of myostatin prevents microgravity-induced loss of skeletal muscle mass and strength
Source: PLoS One. 2020 Apr 21;15(4):e0230818. doi: 10.1371/journal.pone.0230818 (PMC7173869; doi:10.1371/journal.pone.0230818)
Supplement: S1 Table — (DOCX) [file pone.0230818.s001.docx]

| **Gene Name** | **Gene Alias** | **ABI Gene Expression Assay ID** | **Cat#** | **Gene Description** |
| --- | --- | --- | --- | --- |
| Actc1 |  | [Mm01333821_m1](http://www.thermofisher.com/taqman-gene-expression/product/Mm01333821_m1?CID=&ICID=&subtype=ge_all) | 4331182 | Alpha cardiac actin |
| Acvr2b | AcvRIIb | [Mm00431664_m1](http://www.thermofisher.com/taqman-gene-expression/product/Mm00431664_m1?CID=&ICID=&subtype=ge_all) | 4331182 | Activin beta 2 receptor in TGFβ family signaling |
| Alk4 | Acvr1b | [Mm00475713_m1](http://www.thermofisher.com/taqman-gene-expression/product/Mm00475713_m1?CID=&ICID=&subtype=ge_all) | 4331182 | Activin beta 1 receptor in TGFβ family signaling |
| Cfd |  | [Mm00442664_m1](http://www.thermofisher.com/taqman-gene-expression/product/Mm00442664_m1?CID=&ICID=&subtype=ge_all) | 4331182 | Complement factor D (adipsin) |
| Cidec |  | [Mm00617672_m1](http://www.thermofisher.com/taqman-gene-expression/product/Mm00617672_m1?CID=&ICID=&subtype=ge_all) | 4331182 | Cell death inducing DFFA-like effector c |
| Cyr61 |  | [Mm00487498_m1](http://www.thermofisher.com/taqman-gene-expression/product/Mm00487498_m1?CID=&ICID=&subtype=ge_all) | 4331182 | Growth factor inducible early gene |
| Dnajb1 |  | [Mm00444519_m1](http://www.thermofisher.com/taqman-gene-expression/product/Mm00444519_m1?CID=&ICID=&subtype=ge_all) | 4331182 | Dnaj heat shock protein member B1 |
| Fasn |  | [Mm00662319_m1](http://www.thermofisher.com/taqman-gene-expression/product/Mm00662319_m1?CID=&ICID=&subtype=ge_all) | 4331182 | Fatty acid synthase |
| Foxo1 |  | [Mm00490671_m1](http://www.thermofisher.com/taqman-gene-expression/product/Mm00490671_m1?CID=&ICID=&subtype=ge_all) | 4331182 | Forkhead box 1, trans. factor in IGF-I/PI3K/Akt pathway, Smads/ TGFβ complex |
| Fst |  | [Mm00514982_m1](http://www.thermofisher.com/taqman-gene-expression/product/Mm00514982_m1?CID=&ICID=&subtype=ge_all) | 4331182 | Follistatin, binds myostatin and activin |
| Fstl1 |  | [Mm00433371_m1](http://www.thermofisher.com/taqman-gene-expression/product/Mm00433371_m1?CID=&ICID=&subtype=ge_all) | 4331182 | Follistatin like 1 |
| Fzd9 |  | [Mm01206511_s1](http://www.thermofisher.com/taqman-gene-expression/product/Mm01206511_s1?CID=&ICID=&subtype=ge_all) | 4331182 | Frizzled class receptor 9, Wnt signaling |
| Gamt |  | [Mm00487473_m1](https://www.thermofisher.com/taqman-gene-expression/product/Mm00487473_m1?CID=&ICID=&subtype=) | 4331182 | Guanidinoacetate methyltransferase, an N-methyltransferase that catalyzes synthesis of creatine |
| Id1 |  | [Mm00775963_g1](http://www.thermofisher.com/taqman-gene-expression/product/Mm00775963_g1?CID=&ICID=&subtype=ge_all) | 4331182 | Inhibitor of DNA binding 1 |
| Igfbp5 |  | [Mm00516037_m1](https://www.thermofisher.com/taqman-gene-expression/product/Mm00516037_m1?CID=&ICID=&subtype=) | 4331182 | Insulin-like growth factor (IGF) binding protein 5 |
| Itgb5 |  | [Mm00439825_m1](https://www.thermofisher.com/taqman-gene-expression/product/Mm00439825_m1?CID=&ICID=&subtype=) | 4331182 | Integrin binding protein 5, focal adhesion |
| Kcnma1 |  | [Mm01268569_m1](http://www.thermofisher.com/taqman-gene-expression/product/Mm01268569_m1?CID=&ICID=&subtype=ge_all) | 4331182 | Potassium calcium-activated channel subfamily M alpha 1 |
| Mybph |  | [Mm00443928_m1](https://www.thermofisher.com/taqman-gene-expression/product/Mm00443928_m1?CID=&ICID=&subtype=ge_all) | 4331182 | Myosin binding protein H |
| Myf6 | Mrf4 | [Mm00435126_m1](http://www.thermofisher.com/taqman-gene-expression/product/Mm00435126_m1?CID=&ICID=&subtype=ge_all) | 4331182 | Muscle specific transcription factor for differentiation and development |
| Mstn | GDF8 | [Mm01254559_m1](http://www.thermofisher.com/taqman-gene-expression/product/Mm01254559_m1?CID=&ICID=&subtype=ge_all) | 4331182 | Negative regulator of muscle growth, TGFβ signaling |
| Pax7 |  | [Mm01354484_m1](https://www.thermofisher.com/taqman-gene-expression/product/Mm01354484_m1?CID=&ICID=&subtype=) | 4331182 | Paired box gene 7, specification of myogenic satellite cells |
| Ppargc1a | Pgc1a | [Mm01208835_m1](http://www.thermofisher.com/taqman-gene-expression/product/Mm01208835_m1?CID=&ICID=&subtype=ge_all) | 4331182 | Peroxisome prolif activated receptor, γ coact 1a |
| Pitx2 |  | [Mm01316994_m1](http://www.thermofisher.com/taqman-gene-expression/product/Mm01316994_m1?CID=&ICID=&subtype=ge_all) | 4331182 | Paired-like homeodomain transcription factor 2 |
| Rbp4 |  | [Mm00803266_m1](http://www.thermofisher.com/taqman-gene-expression/product/Mm00803266_m1?CID=&ICID=&subtype=ge_all) | 4331182 | Retinol binding protein 4 |
| Retn |  | [Mm00445641_m1](http://www.thermofisher.com/taqman-gene-expression/product/Mm00445641_m1?CID=&ICID=&subtype=ge_all) | 4331182 | Resistin |
| Slc38a2 |  | [Mm00628416_m1](http://www.thermofisher.com/taqman-gene-expression/product/Mm00628416_m1?CID=&ICID=&subtype=ge_all) | 4331182 | Solute carrier 38, member2 |
| Trim63 | MuRF1 | [Mm01185221_m1](http://www.thermofisher.com/taqman-gene-expression/product/Mm01185221_m1?CID=&ICID=&subtype=ge_all) | 4331182 | Muscle ring finger protein, ubiquitin ligase in sk. muscle atrophy |
| Zmynd17 | Mss51 | [Mm01279355_m1](http://www.thermofisher.com/taqman-gene-expression/product/Mm01279355_m1?CID=&ICID=&subtype=ge_all) | 4331182 | Zinc finger family |
