# Supplementary material for: Inhibition of myostatin prevents microgravity-induced loss of skeletal muscle mass and strength
Source: PLoS One. 2020 Apr 21;15(4):e0230818. doi: 10.1371/journal.pone.0230818 (PMC7173869; doi:10.1371/journal.pone.0230818)
Supplement: S2 Table — (DOCX) [file pone.0230818.s002.docx]

**S2 Table**: Gene expression values for Gastronemius, Quadriceps, Soleus and Tibialis anterior muscles from Flight and Ground groups

| Gene | Gastrocnemius | | | | Quadriceps | | | | Soleus | | | | Tibialis Anterior | | | |
| --- | --- | --- | --- | --- | --- | --- | --- | --- | --- | --- | --- | --- | --- | --- | --- | --- |
|  | Grd IgG | Grd YN41 | Flight IgG | Flight YN41 | Grd IgG | Grd YN41 | Flight IgG | Flight YN41 | Grd IgG | Grd YN41 | Flight IgG | Flight YN41 | Grd IgG | Grd YN41 | Flight IgG | Flight YN41 |
| Actc1 | 0.59  0.02 | **1.02**  0.05 | **0.37**  0.03 | **0.79**  0.05 | 0.60  0.03 | 0.65  0.07 | 0.70  0.08 | 1.00  0.11 | 0.03  0.00 | 0.04  0.01 | **0.06**  0.01 | 0.05  0.00 | 0.14  0.01 | **0.31**  0.02 | **0.09**  0.01 | **0.17**  0.01 |
| Acrv2b | 1.25  0.05 | 1.27  0.01 | 1.18  0.04 | 1.28  0.08 | 0.79  0.07 | **0.45**  0.03 | 0.92  0.06 | 0.79  0.05 | 0.73  0.04 | 0.76  0.10 | **1.10**  0.09 | **0.82**  0.06 | 0.87  0.05 | 0.86  0.06 | 0.73  0.05 | 0.67  0.02 |
| Alk4 | 0.57  0.05 | 0.54  0.04 | 0.52  0.04 | 0.60  0.03 | 0.29  0.02 | **0.20**  0.01 | 0.30  0.01 | 0.30  0.02 | 0.14  0.01 | 0.16  0.02 | **0.19**  0.01 | 0.16  0.01 | 0.35  0.03 | 0.30  0.02 | 0.30  0.02 | 0.30  0.01 |
| Cfd | 3.48  0.80 | 3.87  0.60 | 5.69  0.80 | 6.21  0.97 | 133.75  17.07 | 88.79  17.33 | 135.68  14.55 | **74.11**  10.09 | 5.52  1.15 | 3.48  1.05 | 3.61  1.03 | 2.26  0.98 | 40.23  12.73 | 33.92  7.30 | 36.66  8.68 | 33.35  4.91 |
| Cidec | 3.38  0.62 | 3.04  0.49 | 4.51  0.33 | 6.23  0.69 | 75.68  11.94 | 55.39  12.78 | 100.77  21.39 | 72.43  7.09 | 4.91  0.92 | 3.44  0.93 | 2.86  0.63 | 1.44  0.23 | 14.75  1.13 | 23.04  4.20 | 18.62  3.06 | 17.89  2.85 |
| Cyr61 | 0.74  0.04 | **0.50**  0.07 | 0.75  0.08 | **0.49**  0.05 | 0.49  0.04 | **0.30**  0.03 | 0.45  0.05 | 0.37  0.03 | 1.63  0.18 | 1.60  0.19 | 2.30  0.63 | **0.61**  0.06 | 1.19  0.10 | **0.85**  0.12 | 1.44  0.22 | **0.60**  0.04 |
| Dnajb1 | 0.91  0.09 | 0.81  0.06 | 0.72  0.06 | **1.18**  0.13 | 0.70  0.08 | 0.52  0.05 | 0.49  0.04 | **0.85**  0.12 | 1.13  0.18 | 0.98  0.10 | 0.71  0.07 | 1.09  0.15 | 0.72  0.06 | 0.58  0.03 | 0.71  0.07 | 0.76  0.08 |
| Fasn | 0.99  0.16 | 0.86  0.11 | 1.01  0.11 | 0.82  0.10 | 3.95  0.56 | **2.22**  0.24 | 0.61  0.39 | 3.30  0.24 | 0.53  0.04 | 0.59  0.13 | 0.67  0.19 | 0.43  0.08 | 0.23  0.01 | 0.30  0.03 | 0.27  0.04 | **0.13**  0.01 |
| Foxo1 | 0.26  0.02 | 0.22  0.01 | 0.29  0.03 | 0.26  0.02 | 0.23  0.03 | 0.15  0.01 | 0.61  0.39 | 0.20  0.02 | 0.53  0.06 | 0.61  0.11 | 0.51  0.07 | 0.49  0.06 | 0.21  0.02 | 0.19  0.02 | 0.22  0.03 | 0.16  0.01 |
| Fst | 1.93  0.19 | 1.55  0.16 | 2.08  0.10 | **0.98**  0.11 | 1.92  0.14 | **1.42**  0.08 | 1.89  0.13 | **0.93**  0.07 | 3.67  0.18 | 4.26  0.53 | 4.59  0.59 | **2.50**  0.13 | 0.88  0.06 | **0.66**  0.08 | 0.91  0.11 | **0.36**  0.01 |
| Fstl1 | 0.77  0.06 | 0.79  0.06 | 0.62  0.03 | 0.55  0.05 | 0.64  0.04 | 0.66  0.02 | 0.72  0.06 | 0.56  0.05 | 1.06  0.09 | 1.16  0.17 | 0.85  0.18 | 0.44  0.06 | 0.62  0.03 | **0.47**  0.04 | 0.64  0.02 | **0.35**  0.04 |
| Frzd9 | 2.11  0.13 | **2.66**  0.11 | **1.71**  0.12 | 2.12  0.16 | 1.36  0.09 | 1.51  0.08 | 1.64  0.12 | 1.73  0.04 | 5.81  0.30 | **3.89**  0.37 | 4.56  0.54 | 3.89  0.37 | 1.26  0.06 | **9.07**  0.47 | **1.75**  0.10 | 1.56  0.06 |
| Gamt | 1.09  0.07 | **1.64**  0.10 | 0.86  0.08 | **1.34**  0.15 | 0.62  0.02 | **0.77**  0.02 | **0.76**  0.03 | **0.90**  0.01 | 0.23  0.02 | 0.32  0.04 | 0.23  0.02 | 0.22  0.02 | 0.58  0.06 | 0.72  0.06 | 0.50  0.05 | 0.49  0.03 |
| Id1 | 0.80  0.12 | 0.71  0.08 | 0.55  0.05 | 0.65  0.08 | 0.51  0.06 | 0.51  0.06 | 0.42  0.03 | 0.46  0.05 | 2.19  0.61 | 1.55  0.20 | 0.71  0.15 | 0.36  0.07 | 0.80  0.09 | 0.59  0.04 | 0.69  0.09 | 0.66  0.08 |
| Igfbp5 | 0.90  0.11 | 1.18  0.09 | 0.79  0.08 | **1.28**  0.10 | 0.51  0.07 | 0.39  0.04 | 0.54  0.04 | 0.64  0.05 | 0.43  0.07 | 0.40  0.04 | 0.58  0.09 | 0.46  0.04 | 0.38  0.04 | 0.50  0.04 | 0.35  0.02 | **0.53**  0.03 |
| Itgb5 | 1.24  0.06 | **0.93**  0.04 | 1.20  0.06 | **0.92**  0.05 | 1.01  0.06 | **0.63**  0.03 | 0.95  0.03 | **0.65**  0.02 | 1.37  0.06 | 1.54  0.19 | 1.25  0.10 | 1.10  0.13 | 1.09  0.02 | **0.84**  0.02 | 1.11  0.07 | **0.72**  0.04 |
| Kcnma1 | 0.97  0.09 | 1.05  0.12 | 0.99  0.14 | 1.18  0.21 | 0.61  0.06 | 0.50  0.04 | 0.61  0.39 | 0.87  0.09 | 6.48  0.23 | 7.46  1.32 | **3.76**  0.44 | 2.94  0.16 | 0.49  0.04 | 0.44  0.02 | 0.42  0.03 | 0.51  0.06 |
| Mybph | 0.49  0.03 | **0.62**  0.03 | **0.37**  0.02 | **0.68**  0.06 | 0.45  0.04 | 0.41  0.03 | 0.49  0.03 | 0.58  0.03 | 0.01  0.00 | 0.00  0.00 | 0.03  0.01 | 0.01  0.00 | 0.18  0.02 | **0.29**  0.03 | 0.14  0.04 | 0.19  0.01 |
| Myf6 | 0.52  0.02 | 0.59  0.03 | 0.55  0.10 | 0.56  0.05 | 0.44  0.03 | 0.42  0.04 | 0.61  0.39 | 0.49  0.03 | 0.65  0.06 | 0.89  0.14 | 0.80  0.14 | 0.55  0.05 | 0.41  0.03 | 0.47  0.03 | 0.41  0.04 | 0.39  0.02 |
| Myostatin | 0.63  0.04 | **0.74**  0.03 | 0.64  0.05 | **0.89**  0.05 | 0.52  0.05 | 0.53  0.04 | 0.67  0.06 | 0.70  0.05 | 0.01  0.00 | 0.01  0.00 | **0.02**  0.00 | 0.02  0.00 | 0.31  0.02 | 0.35  0.03 | 0.43  0.05 | 0.39  0.01 |
| Pax7 | 1.49  0.13 | 1.48  0.12 | 1.71  0.21 | 1.41  0.13 | 1.36  0.16 | **0.86**  0.15 | 1.16  0.13 | 0.91  0.09 | 4.98  0.54 | 5.40  0.96 | 5.88  0.63 | 4.44  0.59 | 1.11  0.09 | 0.99  0.08 | 1.15  0.11 | **0.68**  0.04 |
| Pitx2 | 0.61  0.03 | 0.56  0.02 | 0.73  0.06 | 0.71  0.06 | 0.61  0.04 | 0.53  0.02 | **0.94**  0.09 | **0.63**  0.03 | 2.61  0.16 | 3.05  0.41 | 2.64  0.15 | 2.46  0.15 | 1.65  0.07 | 1.48  0.14 | 1.72  0.06 | **1.48**  0.04 |
| Ppargc1a | 0.64  0.02 | 0.65  0.03 | **0.54**  0.03 | **0.64**  0.05 | 0.33  0.04 | 0.30  0.03 | 0.43  0.04 | 0.38  0.03 | 0.79  0.09 | 1.34  0.23 | 1.03  0.16 | 0.86  0.12 | 0.63  0.03 | 0.65  0.04 | 0.60  0.06 | 0.51  0.03 |
| Rbp4 | 0.85  0.22 | 0.54  0.10 | 0.66  0.11 | **0.34**  0.03 | 2.12  0.23 | 1.60  0.33 | 2.61  0.32 | **1.31**  0.09 | 0.46  0.08 | 0.47  0.09 | 0.39  0.12 | 0.31  0.16 | 0.22  0.02 | 0.21  0.02 | 0.18  0.02 | **0.10**  0.01 |
| Retn | 1.07  0.16 | **0.64**  0.07 | 1.29  0.23 | **0.46**  0.06 | 4.54  0.46 | **2.96**  0.37 | 5.79  0.63 | **2.70**  0.27 | 0.12  0.02 | 0.09  0.03 | 0.09  0.02 | 0.05  0.01 | 0.19  0.01 | 0.19  0.03 | **0.12**  0.01 | **0.06**  0.00 |
| Slc38a2 | 0.44  0.03 | 0.44  0.04 | **0.74**  0.10 | 1.05  0.18 | 0.61  0.09 | 0.45  0.06 | 0.86  0.10 | 0.72  0.07 | 1.75  0.22 | 1.63  0.10 | 1.48  0.23 | 1.04  0.07 | 0.55  0.02 | 0.71  0.07 | 0.54  0.03 | 0.53  0.03 |
| Trim63 | 0.89  0.06 | 0.89  0.04 | 0.78  0.06 | 0.81  0.02 | 0.68  0.04 | 0.56  0.04 | 0.64  0.05 | 0.59  0.03 | 0.75  0.03 | 1.02  0.12 | **1.01**  0.08 | **0.80**  0.05 | 0.61  0.03 | 0.54  0.01 | 0.60  0.05 | 0.55  0.01 |
| Zymd17 | 1.50  0.21 | **0.49**  0.03 | 1.94  0.26 | **0.74**  0.06 | 0.51  0.04 | **0.25**  0.02 | 0.66  0.06 | **0.26**  0.02 | 0.00  0.00 | 0.00  0.00 | **0.01**  0.00 | **0.00**  0.00 | 0.15  0.02 | **0.05**  0.01 | 0.21  0.03 | **0.05**  0.01 |

Mean gene expression values shown with sem variance below. **Bold** indicates significance to respective IgG control or for Flight IgG group, to Grd IgG control, p<0.05**.**
